# Elucidating the Effect of Antecedents on Consumers**’** Green Purchase Intention: An Extension of the Theory of Planned Behavior

**DOI:** 10.3389/fpsyg.2020.01433

**Published:** 2020-07-24

**Authors:** Athapol Ruangkanjanases, Jun-Jer You, Shih-Wen Chien, Yin Ma, Shih-Chih Chen, Ling-Chi Chao

**Affiliations:** ^1^Chulalongkorn Business School, Chulalongkorn University, Bangkok, Thailand; ^2^Department of Exercise and Health Sciences, University of Taipei, Taipei City, Taiwan; ^3^Department of Intelligent Commerce, National Kaohsiung University of Science and Technology, Kaohsiung City, Taiwan; ^4^School of Philosophy and Sociology, Lanzhou University, Lanzhou, China; ^5^Department of Information Management, National Kaohsiung University of Science and Technology, Kaohsiung City, Taiwan; ^6^Department of Information Management, Tatung University, Taipei City, Taiwan

**Keywords:** theory of planned behavior, green consumption, green products purchase intention, green food, structural equating modeling, purchase intention

## Abstract

In this study, the extension of theory of planned behavior was developed to evaluate the effects of antecedents that influence consumers’ intention to buy green products. The effect of nine determinants (i.e., individual benefits, social benefits, willingness-to-pay, environmental responsibility, e-word-of-mouth, values, self-competence, convenience, and environmental literacy) of the green wave on Taiwanese consumers was empirically tested by examining their perception of social responsibility through theory of planned behavior. Except for consumer subjective norms, the remaining factors exhibited significant positive correlations with the planned behavior, implying that the voluntary participation aspect of the green wave is considerably more critical than its mandatory social pressure. To diffuse this green wave more effectively, the Taiwanese government should encourage consumers to easily capture the detailed impact of the green wave on the society and allow consumers to use word-of-mouth marketing for the creation of relational value to improve their quality of life.

## Introduction

Following the implementation of the United Nations Framework Convention on Climate Change in 1994, diverse efforts have been made in the public and private sectors worldwide to achieve sustainable global development. At the 2015 United Nations Climate Change Conference in Paris, the twenty-first session of the Conference of the Parties agreed to limit the increase temperature to below 2°C above preindustrial levels in the global average. Moreover, the delegates agreed to continue their efforts to limit the temperature rise to 1.5°C above preindustrial levels. These agreements indicated that temperature-control efforts would greatly reduce the risks of climate change. To achieve the objectives of United Nations Framework Convention on Climate Change, 195 member countries must make clear the measureable efforts for global sustainable development. In particular, the public–private partnership in sustainable development is crucial for achieving this objective, including in Taiwan (Choi et al., 2016).

Taiwan is one of the most dangerous areas in the world from an environmental perspective (Tsai and Chen, 2011). More than 70% of the land area and population in Taiwan face the threats of more than three types of natural disasters simultaneously (World Bank and Columbia University, 2005). Such concerns are reflected in online activities and have led to diverse environmental movements in Taiwan. In October 2013, many netizens claimed that several soy sauce companies used hydrochloric acid to decompose soybean sauce, thus resulting in higher risks of kidney damage, cancer, and many other diseases. The Taiwanese society was traumatized by this terrible chemical disaster and hence emphasized food security concerns. Global warming, waste water and pollution, loss of species, the destruction of the ozone layer, and other environmental concerns have become key threats to sustainable human existence globally, particularly in Taiwan (Tanner and Wölfing Kast, 2003). Thus, the turmoil of the sustainable, or green wave, the third revolution in human history, has been affecting Taiwan.

Classical economies were defined by the “self-sufficiency paradigm” of these societies. Because supply creates its own demand, classical economies did not experience oversupply problems (Sowell, 2015). However, the industrial revolution introduced large-scale mass production led by companies with the new paradigm of “economies of scale.” The more a company produces, the lower the unit cost is, resulting in a permanent oversupply problem of current societies. At the beginning of the industrialization period, companies and governments solved their oversupply problem through overseas colonies, but this imperialism was unsustainable because of the claims from latecomers such as Germany, Russia, and Japan. Because of the ever-increasing oversupply problems associated with energy-intensive economic structures, this company-led industrial revolution introduced excessive energy and resource consumption, resulting in global warming and the extreme bipolarization of societies and countries.

Therefore, after the 300-year industrialization period, the green wave revolution emerged with the new paradigm of “value creation based on the network management.” Instead of competitive equilibrium or optimum of a single economic entity, which were the core paradigms of traditional economics and business management, the harmonized cooperation among many partners to improve quality of life has become the core objective in the green or sustainable wave. Rather than a strategy to obtain the largest portion of a pizza, the paradigm of the green wave is reflected by cooperative partnership to enlarge the size of the pizza to benefit all partners. Initially the emphasis of the green wave on the environmental protection was excessively strong; however, this emphasis has shifted toward harmonizing economic development for the needs of current and future generations. As environmental awareness among these new proactive consumers has increased, the concept of environmental protection has gradually become an invisible pressure on all economic entities, and this new environmental wave has slowly changed consumers’ daily consumption behaviors. The behaviors of these green consumers are termed green consumption. If customers want to contribute to environmental protection, the simplest approach is purchasing green products. Green products should be defined on the basis of the wider perspective of green supply chain management, which aims to reduce pollution and resource consumption. This includes the acquisition of environmentally friendly raw materials, environmentally friendly manufacturing and distribution, energy reduction, and environmentally friendly disposal, including recycling, biodegradable disposal management, clean energy and water use, packaging reduction, and limiting toxic byproducts (Nimse et al., 2007). The actions of manufacturing companies and regulatory authorities are based on the strong but invisible pressure of green consumers, who are the drivers of all economic activities. Therefore, the behavior yielded by consumers’ attitudes toward the green wave is crucial for the successful economic transformation of companies and governments.

To understand the social transformation toward the green wave, the nine determinants that promote green consumption were analyzed in this research through the theory of planned behavior. The rest of the paper is organized as follows. Section “Literature Review and Hypothesis Development” reviews the previous studies and then proposed hypotheses and the research model. Section “Research Methodology and Sample Structure” presents the empirical analysis and its results and implications. Finally, Section “Data Analysis Result” concludes several summary and strategic suggestions.

## Literature Review and Hypothesis Development

In this section, through a comparative analysis of previous studies, theory of planned behavior, including diverse sustainable factors, is introduced as a methodological basis. Hypotheses are established on the basis of common arguments.

### Theory of Planned Behavior

Theory of planned behavior, which was derived from the theory of reasoned action (Fishbein and Ajzen, 1977), connects the beliefs and behaviors of potential consumers. Both models are based on the premises that individuals make logical, reasoned decisions to engage in specific behaviors by evaluating the information available to them. The theory states that consumers’ attitudes toward their realized behavior are based on their subjective behavioral belief in the product or service and their cognitive evaluation of the possible results; these attitudes may be affected by the subjective norms derived from the more objective specifications of the faith on the consumption and obedience motivation of the consumer. Subjective norms are a type of perceived behavioral control mechanism of consumption. Combined with consumers’ attitudes, subjective norms form individuals’ behavioral intentions and their resulting behaviors. More specifically, consumer attitudes could be defined as the positive or negative evaluation of the self-expression of particular behaviors. This conceptual framework measures the extent to which the performance of consumers’ behavior is positively or negatively associated with their purchase intentions. In this study, consumer attitudes were defined through consumers’ positive evaluations of environmentally friendly fabricated products.

Subjective norms were defined as individuals’ perception of a particular behavior, which are strongly impacted by the judgments of other individuals such as parents, friends, spouses, and teachers. In this study, subjective norms were defined as consumers’ perception of environmentally friendly products in the face of social pressure, resulting in support for or opposition to green products, which negatively affects the environment. If consumers evaluate the suggested behavior regarding a product or service as positive (attitude), and if they consider this attitude as significant to others for them to perform the behavior more appropriately (subjective norm), higher intentions are obtained (motivations); thus, consumers are more likely to engage in this behavior. Many studies have demonstrated a high correlation between the consumers’ attitudes, the surrounding subjective norms of behavioral intention, and the purchase behavior itself (e.g., Liao et al., 2007; Khalifa and Shen, 2008; Chen et al., 2009; Lu et al., 2009; Nasri and Charfeddine, 2012).

**FIGURE 1:**
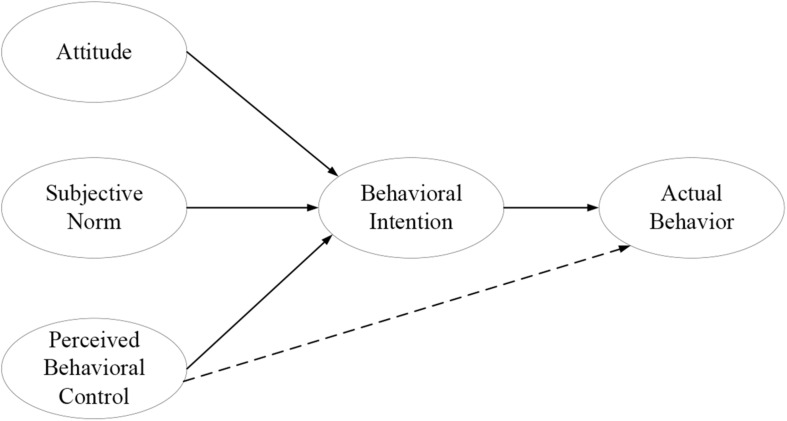
Theory of planned behavior.

Ramayah et al. (2009) extended theory of planned behavior by introducing an additional factor of perceived behavioral control to the purchase intention, which is defined as an individual’s perceived easiness or difficulty in performing the behavior. Perceived behavioral control is determined according to all the accessible control beliefs. If consumers receive more information related to the green characteristics on a product and thus have higher perceived behavioral control, then they have a greater purchase intention for environmentally friendly products. Intention is defined as an indication that a person is ready to perform a certain act and is considered as the direct antecedent of behavior. In this study, intention was defined as the possibility that consumers will choose to purchase the environmentally friendly products. The basic framework of theory of planned behavior is presented in Figure 1.

### Hypotheses and Model Development

In this section, the development of the logical factors or determinants on the purchase intention of theory of planned behavior model shown in Figure 1 is described in detail. Among the relevant arguments, individual as well as social benefits are defined as consumers’ cognitive potential benefits of their attitude toward the purchase of environmentally friendly products. If a product uses an environmentally friendly coating to avoid harmful gases, consumers can alter their behavioral intention through personal interest, attitude, and psychological physiology for this green product (Bhattacherjee, 2000; Kotler and Keller, 2006). This study focused on the effect of the green wave paradigm shift from the competitive solution of profit-maximization on the production of environmentally friendly products. Therefore, the following hypothesis could be a valuable initial approach to consumers’ attitude shift toward the green wave.

H1: Individual benefits have a positive influence on consumer attitudes.

Consumers obtain not only individual benefits from the purchase of environmentally friendly products but also social benefits. If a green product that includes recycled materials could reduce environmental damage, then most consumers would feel that they could contribute to social responsibility to preserve the environment by purchasing this product. Therefore, social benefits affect attitude. Manaktola and Jauhari (2007) emphasized that consumers with a strong social responsibility hold relatively positive attitudes toward environmentally friendly products. Thus, we propose the following hypothesis.

H2: Social benefits have a positive influence on consumer attitudes.

Because the cognition of green consumption has been widely accepted, consumers are willing to spend more money to buy green products. Ozanne and Vlosky (1997) found that US consumers were willing to pay 4.4–18.7% higher prices for environmentally friendly certified wooden products. Moreover, according to Rushmore (1993), US tourists are willing to pay 8.5% more for green hotels. Hansla et al. (2008) revealed that consumers are also willing to pay more for green electricity such as that generated from renewable sources. Thus, willingness to pay is strongly positively related to consumers’ attitude regarding the purchase intentions for environmentally friendly products. Therefore, consumers have a positive attitude toward green products regardless of price level. According to Abeliotis et al. (2010), most consumers are willing to spend more money for products that have less impact on the environment, which indicates a positive attitude regarding purchase intention. Based on these arguments, the third hypothesis of willingness to pay is proposed.

H3: Willingness to pay has a positive influence on consumer attitudes.

Environmental responsibility is a type of consumer awareness of the responsibility to maintain the integrity of the environment, and it is indicated by proactive caring for the environment. Consumers have a sense of creating values when they engage in environmental protection. Indicated that responsibility should explain individuals’ self-disciplined conduct and encourage consumers to make ethical decisions, plan ahead, try their best, and set a good example. Pearce and Gregersen (1991) used the variable of responsibility to indicate workers’ perceptions. The responsibility of workers is to care for others, including customers and colleagues, and this is a type of mandatory feeling for them to help others. Bansal (2011) suggested that consumers with high environmental awareness as subjective norms exhibit preferences for green products. Therefore, the following hypothesis on environmental responsibility is established.

H4: Environmental responsibility has a positive influence on subjective norms.

Electronic word-of-mouth can be considered as a type of invisible constraint on certain group members through the relational sharing of information; thus, it can serve as the basis for the subjective norms of that group. Hanson and Kalyanam (2007) analyzed electronic word-of-mouth and found that the consumers face the social pressure to purchase environmentally friendly products because of the broad discussion and consensus on the Internet about the influence of the green wave. According to Bickart and Schindler (2001), the higher the credibility of Internet goes by word-of-mouth, the stronger its influence on consumption is. Prior research also highlighted that electronic word-of-mouth affects subjective norms, resulting in a more positive attitude regarding behavioral intention (e.g., Jalilvand and Samiei, 2012; Fu et al., 2015; Husin et al., 2016). Thus, the fifth hypothesis on electronic word-of-mouth is proposed.

H5: Electronic word-of-mouth has a positive influence on subjective norms.

Subjective values that emerge from environmental protection activities could serve as social subjective norms. Schwartz (1992) emphasized values as the preference for a certain behavior or way of life of a person or social group with enduring beliefs. Manaktola and Jauhari (2007) concluded that consumers with higher altruistic values are very likely to be green consumers. Therefore, the following hypothesis on values is proposed.

H6: Perceived value has a positive influence on subjective norms.

In the context of environmentally friendly products, self-competence is defined as consumers having sufficient basic knowledge, skills, and income to choose and purchase the products. Ajzen (1991) noted that perceived behavioral control includes self-efficacy or self-competence to facilitate the surrounding conditions. The higher consumers’ self-competence is, the more they are likely to implement the three Rs (reduce, recycle, reuse) for environmental conservation and protection (Teisl and O’Brien, 2003; Thapa and Graefe, 2003; Fraj and Martinez, 2006). Thus, we propose the following hypothesis.

H7: Self-competence has a positive influence on perceived behavioral control.

To promote environmentally friendly products, consumer convenience for purchasing green products will enhance their purchase intentions (Chiu, 2009). Convenience for purchasing products or obtaining information is very perceptive. Yoon and Kim (2007) indicated that consciousness of convenience is an external variable that affects consumer behavior. Hossain and Prybutok (2008) indicated that consciousness of convenience affects the intention to use. Therefore, the following hypothesis is proposed.

H8: Convenience has a positive influence on perceived behavioral control.

Roth (1992) argued that environmental literacy is crucial for consumers to consider environmental concerns. According to subject-oriented teaching study conducted by Culen and Volk (2000), students’ literacy on environmental problems is an important determinant because their awareness of the relevant and perceptive behaviors is positively related to perceived behavioral control (McBeth and Volk, 2009; Bansal, 2011). Thus, the following hypothesis is proposed.

H9: Environmental literacy has a positive influence on perceived behavioral control.

Theory of planned behavior posits the relations between beliefs, attitudes regarding purchase intentions, and the resulting behavior. According to this model, consumer attitudes toward behavior are determined by their accessible beliefs about environmentally friendly production, where beliefs are defined as the subjective probabilities that the behavior will yield certain outcomes. Specifically, the evaluation of each outcome brings the attitude in direct proportion to the person’s subjective probability that the behavior produces the outcome in question (Kalafatis et al., 1999; Ajzen, 2002; Chen et al., 2009; Han and Kim, 2010; Han et al., 2010; Cheon et al., 2012; Jekauc et al., 2015; Yadav and Pathak, 2017; Zampetakis et al., 2017; Verma and Chandra, 2018; Sun et al., 2019). Subjective norms are an individual’s perceptions of a particular behavior, which are strongly influenced by the judgments of others (Trumbo and O’Keefe, 2005; Chung, 2016; Yadav and Pathak, 2017; Verma and Chandra, 2018). Ajzen (1985) argued that perceived behavioral control is determined by the total set of accessible control beliefs. Therefore, the following hypotheses are proposed.

H10: Attitude has a positive influence on purchase intention.
H11: Subjective norms have a positive influence on purchase intention.
H12: Perceived behavioral control has a positive influence on purchase intention.

**FIGURE 2:**
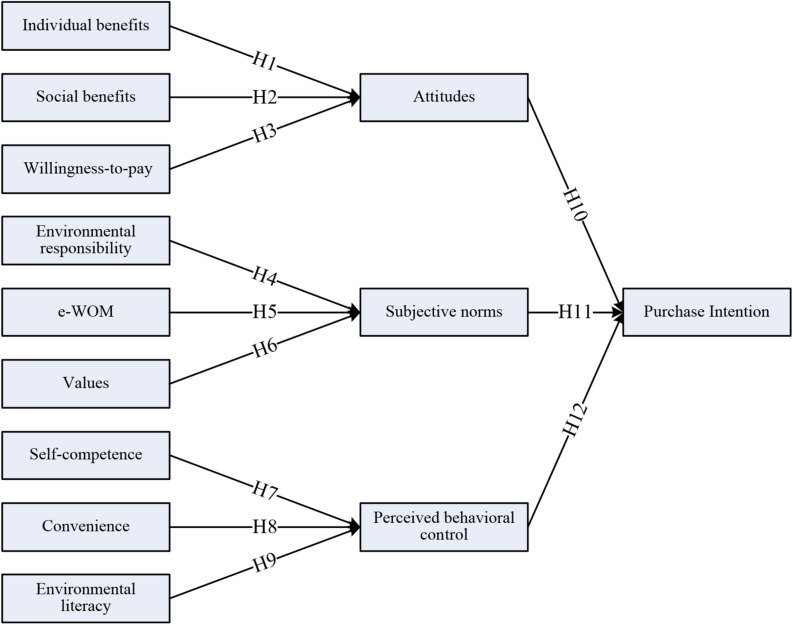
Research model.

The research model, which is based on these hypotheses and theory of planned behavior model, is illustrated in Figure 2.

## Research Methodology and Sample Structure

A questionnaire survey was employed to collect data. The measurement items, research framework, and operational definitions were based on the established hypotheses and other studies. Although most of the measurement items were from existing studies to establish the content validity (as shown in Appendix), we conducted a pilot test with master students to validate the measurement items. Based on the feedback of the pilot test, we revised the wording of the measurement items.

Participants with green products usage experience were recruited by visiting service and marketing relevant classes and asking for volunteers to finish surveys in the spring and summer quarters of 2014 in Taiwan. Then, the data collection launched as a convenience sampling and finally broadened a snowball sampling. A total of 389 samples were collected after our survey of this study. After eliminating the repetitive responses by checking the screening of e-mail and IP addresses, the final valid sample is composed of 353 participants.

To analyze the proposed model, the partial least squares approach was used for structural equation modeling with path and regression analysis. SPSS 18.0 and Smart Partial Least Squares 2.0 were employed to perform the partial least squares analysis (Ringle et al., 2005). We referred to previous studies to formulate the operational definitions and measurement items for all the variables. Finally, 410 questionnaires were collected, 353 of which were valid. To clarify the sample characteristics, Table 1 shows the demographics of the participants. Male and female respondents constituted 51 and 49% of the sample, respectively. Furthermore, the majority of respondents were aged 26–45 years (83.8%). The largest group was college graduates (66.3%) and the second largest was educated to master’s degree level or above (24.4%). Most of the respondents were employed in the high-tech industry (24.4%), followed by the manufacturing sector (17%). Ninety percent of the respondents used the Internet for more than 60 min. All data samples appropriately reflected the societal structure of Taiwan, and the respondents appeared to be familiar with the green wave. The analysis of the outer and inner models on the basis of these samples is presented in the following sections.

**TABLE 1:** Sample demographics.

Characteristics	Description	Frequency	Percent(%)
Gender	Female	173	49.0%
	Male	180	51.0%
Age	Under 25 years old	9	2.5%
	26∼30 years old	35	9.9%
	31∼35 years old	91	25.5%
	36∼40 years old	101	28.6%
	41∼45 years old	70	19.8%
	46∼50 years old	24	6.7%
	51 years old or above	23	6.3%
Education level	Primary school or lower degree	9	2.5%
	Middle school	10	2.8%
	High school certificate	14	4.0%
	Junior college	75	21.3%
	Undergraduate degree	159	45.0%
	Master or higher degree	86	24.4%
Job	Soldier, civil servants, teachers	16	4.5%
	Financial	37	10.5%
	Manufacturing	60	17.0%
	High-tech industries	86	24.4%
	Healthcare	9	2.5%
	Service industries	88	25%
	Freelance	13	3.7%
	Housekeeper	18	5.1%
	Other	26	7.3%

## Data Analysis Results

### Outer Model Analysis

This study followed a two-stage approach to execute data analysis (Anderson and Gerbing, 1988). The first stage is to measure the construct validity of the outer model (measurement model), and then the proposed casual model and research hypotheses were assessed by inner model analysis.

Outer model is defined as the relations between latent constructs and indicators in the partial least squares method. Table 2 tabulates the factor loads and reliability testing results of all the construct items. The composite reliability values of all constructs were ≥0.7, suggesting that the constructs had satisfactory reliability. The convergent validity and discriminant validity tests were performed to assess factors’ construct validity. According to Fornell and Larcker (1981), the requirements for convergent validity would be satisfied if the factor loads of the indicators are more than 0.5; the average variance extracted (AVE) is greater than 0.5; and the reliability is over 0.7. Table 2 indicates that all the constructs satisfied the criteria suggested by Fornell and Larcker (1981), indicating their favorable convergent validity. In addition, a construct features discriminant validity if the square root of the AVE is greater than the correlation coefficient. Tables 2, 3 present the discriminant validity of the constructs. All dimensions had AVE and composite reliability that exceeded the aforementioned cutoff values, which suggests a favorable convergent validity. Furthermore, because the square root of the AVE for each construct exceeded the correlation share among the constructs in our proposed model, discriminant validity was also satisfied (Fornell and Larcker, 1981; Hair et al., 2016).

**TABLE 2:** Reliability analysis and convergent validity.

Dimensions	Research variables	Factor loadings (>0.5) *T*-value (>2)	AVE (>0.5)	Composite reliability (>0.7)
Individual benefits	IB1	0.74 (17.21)	0.51	0.76
	IB2	0.74 (14.08)		
	IB3	0.66 (9.04)		
Social benefits	SB1	0.82 (20.26)	0.77	0.91
	SB2	0.90 (54.83)		
	SB3	0.90 (64.88)		
Willingness-to-pay	WTP1	0.91 (80.11)	0.78	0.91
	WTP2	0.88 (42.14)		
	WTP3	0.85 (37.30)		
Consumer attitudes	CA1	0.95 (99.73)	0.90	0.96
	CA2	0.95 (100.10)		
	CA3	0.95 (118.26)		
Environmental responsibility	ER1	0.67 (9.50)	0.57	0.80
	ER2	0.66 (9.09)		
	ER3	0.92 (40.89)		
e-WOM	EWOM1	0.86 (39.06)	0.72	0.88
	EWOM2	0.87 (50.00)		
	EWOM3	0.81 (29.64)		
Values	V1	0.93 (90.99)	0.81	0.93
	V2	0.93 (96.73)		
	V3	0.84 (25.76)		
Subjective norms	SN1	0.88 (41.93)	0.84	0.94
	SN2	0.94 (88.44)		
	SN3	0.94 (125.89)		
Self-competence	SC1	0.84 (58.28)	0.67	0.86
	SC2	0.80 (22.26)		
	SC3	0.81 (25.74)		
Convenience	CON1	0.78 (18.64)	0.68	0.86
	CON2	0.84 (26.27)		
	CON3	0.85 (51.13)		
Environmental literacy	EL1	0.79 (20.65)	0.61	0.82
	EL2	0.70 (11.97)		
	EL3	0.84 (39.53)		
Perceived behavior control	PBC1	0.81 (26.83)	0.63	0.83
	PBC2	0.69 (11.56)		
	PBC3	0.87 (48.26)		
Intention	INT1	0.94 (90.25)	0.83	0.94
	INT2	0.94 (111.25)		
	INT3	0.86 (25.48)		

**TABLE 3:** Correlation matrix.

	IB	SB	WTP	CA	ER	EWOM	V	SN	SC	CON	EL	PBC	INT
IB	**0.72**												
SB	0.46	**0.88**											
WTP	0.35	0.41	**0.88**										
CA	0.49	0.61	0.58	**0.95**									
ER	0.38	0.55	0.38	0.53	**0.76**								
EWOM	0.48	0.43	0.47	0.64	0.52	**0.85**							
V	0.40	0.49	0.80	0.67	0.47	0.64	**0.90**						
SN	0.37	0.37	0.67	0.60	0.44	0.61	0.75	**0.92**					
SC	0.46	0.35	0.57	0.52	0.42	0.69	0.66	0.63	**0.82**				
CON	0.36	0.49	0.47	0.58	0.55	0.61	0.57	0.55	0.62	**0.82**			
EL	0.33	0.55	0.38	0.52	0.55	0.44	0.45	0.38	0.41	0.59	**0.78**		
PBC	0.41	0.59	0.57	0.65	0.60	0.61	0.63	0.60	0.64	0.70	0.65	**0.79**	
INT	0.35	0.54	0.50	0.65	0.61	0.53	0.59	0.53	0.50	0.64	0.59	0.72	**0.91**

*IB, individual benefits; SB, social benefits; WTP, willingness to pay; CA, consumer attitudes; ER, environmental responsibility; EWOM, eWOM; V, values; SN, subjective norms; SC, self-competence; CON, convenience; EL, environmental literacy; PBC, perceived behavior control; INT, intention.*

Regarding the overall quality of the proposed model, we computed the Goodness of Fit (GoF) following the formula by Tenenhaus et al. (2005). The GOF is calculated as:

$$\text{GOF}=\,\sqrt{\bar{C\,o\,m\,m\,u\,n\,a\,l\,i\,t\,y}\times \bar{R^{2}}}=\sqrt{0.717\times 0.680}=0.698$$

GoF for the outer model as a whole indicate the fitness of the proposed model explains the empirical data. According to above result, GoF is 0.698 which exceeds the cut-off criterion of 0.36 for a large effect size (Wetzels et al., 2009). Therefore, our empirical data concluded the proposed model has an appropriate overall fit, and concede us to conclude our proposed model performs well compared to the baseline values defined above.

### Inner Model Analysis

**FIGURE 3:**
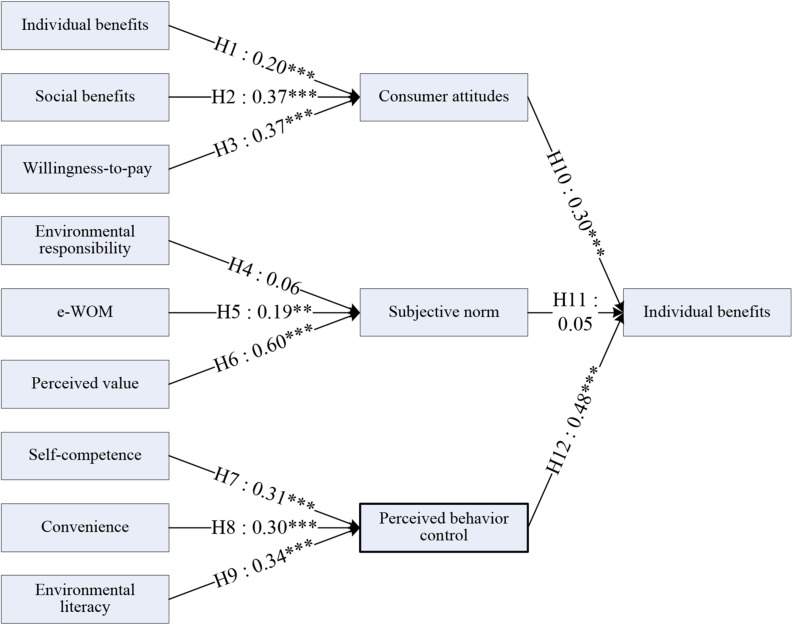
Results of the inner model.

In the PLS approach, the inner model is formed of the path structures between constructs. The hypothesis testing results for the inner model with path coefficients and their significance levels are illustrated in Figure 3 and Table 4. The direct influence of E on H and H on M was not significant, and thus H4 and H11 were not supported. Apart from H4 and H11, the remaining 10 hypotheses formulated in this research were supported at a significance level of 95%. The empirical results revealed that consumers’ attitude and perceived behavioral control strongly supported the purchase intention for green products, whereas the effect of subjective norms obtained from the societal background on the purchase intention was not strong. Therefore, although the Internet diffuses awareness of the green wave, global warming, and climate change, consumers do not strongly believe the information and discussions obtained through the Internet. In particular, consumers do not understand the green wave as a type of environmental responsibility; thus, this type of social norm does not affect consumers’ purchase intentions. The public pressure for the green wave is not important for individuals’ decision-making, an effect that is unique to Taiwan. By contrast social subjective norms have a very strong effect on individual decision-making in Japanese culture.

**TABLE 4:** Hypotheses testing result.

Hypothesis	Standardized path coefficient	*t*-value	Result
H1: Individual benefits have a positive influence on consumer attitudes.	0.195***	3.558	Supported
H2: Social benefits have a positive influence on consumer attitudes.	0.366***	5.639	Supported
H3: Willingness to pay has a positive influence on consumer attitudes.	0.366***	5.475	Supported
H4: Environmental responsibility has a positive influence on subjective norms.	0.057	1.155	Not supported
H5: e-WOM has a positive influence on subjective norms.	0.193**	2.759	Supported
H6: Perceived value has a positive influence on subjective norms.	0.604***	8.281	Supported
H7: Self-competence has a positive influence on perceived behavioral control.	0.313***	6.083	Supported
H8: Convenience has a positive influence on perceived behavioral control.	0.301***	5.352	Supported
H9: Environmental literacy has a positive influence on perceived behavioral control.	0.340***	8.481	Supported
H10: Attitude has a positive influence on purchase intention.	0.300***	3.902	Supported
H11: Subjective norms have a positive influence on purchase intention.	0.057	0.784	Not supported
H12: Perceived behavioral control has a positive influence on purchase intention.	0.487***	6.544	Supported

** *p*-value < 0.01 and *** *p*-value < 0.001.

The estimates of the R-Square exceeded the respective cutoffs proposed in previous studies, confirming that the measurement model exhibited a reasonable fit with the data collected. Table 5 examines the influences of each construct, including direct effects, indirect effects, or both. The path coefficient from a predictor variable to a response variable assesses the direct effect, and the product of the coefficients from a predictor variable through a mediator variable measures the indirect effect.

**TABLE 5:** R-square for the endogenous constructs.

Endogenous construct	*R*^2^ (>0.36)
Consumer attitudes	0.69
Subjective norms	0.71
Perceived behavior control	0.63
Intention	0.69

In contrast to the rejection of the relationship between environmental responsibility and purchase intention, electronic word-of-mouth and perceived value had positive effects on the purchase intention with subjective norms as a mediator. This implies that even if the social pressure is unimportant to individual decisions, the recommendations from intimate social relations are still supportive. Therefore, the public–private partnership is critical to the sustainable transformation of the Taiwanese economy. Instead of mandatory or pseudo pressure by the public for environmental protection, consumers should voluntarily participate in the environmental movement, and the government should implement nudge policies by managing sustainable networks for consumers to voluntarily share information and experiences of environmental protection. For this purpose, the public–private partnership is crucial in Taiwan and other Asian countries such as China and Korea, where the role of the government is culturally limited.

### Testing of Mediation Effects

This study adopted Sobel, Aroian, and Goodman tests to analyze the mediation effect of three behavioral factors of the consumers, namely their attitude, subjective norms, and perceived behavioral control. A significant mediation effect between consumers’ attitude and perceived behavioral control is present if the absolute value of *z* is greater than 1.96 (Aroian, 1947; Goodman, 1960; Sobel, 1982). The direct influence of environmental responsibility on consumers’ attitude, perceived behavioral control, and subjective norms was insignificant; thus, mediation effects were not necessary to assess. The remaining constructs also underwent mediation effect testing, and the results are tabulated in Table 6.

**TABLE 6:** Mediation effects testing.

Equation	Relationship	*t*-value	Sobel test	Aroian test	Goodman test
IB → CA → INT	IB → CA	3.5575	2.63	2.58	2.68
	CA → INT	3.9020			
SB → CA → INT	SB → CA	5.6393	3.21	3.18	3.24
	CA → INT	3.9020			
WTP → CA → INT	WTP → CA	5.4755	3.18	3.14	3.21
	CA → INT	3.9020			
SC → PBC → INT	SC → PBC	6.0825	4.46	4.43	4.48
	PBC → INT	6.5436			
CON → PBC → INT	CON → PBC	5.3524	4.14	4.11	4.17
	PBC → INT	6.5436			
EL → PBC → INT	EL → PBC	8.4808	5.18	5.16	5.20
	PBC → INT	6.5436			

*IB, individual benefits; SB, social benefits; WTP, willingness-to-pay; CA, consumer attitudes ER, environmental responsibility; EWOM, eWOM; V, values; SN, subjective norms; SC, self-competence; CON, convenience; EL, environmental literacy; PBC, perceived behavior control; INT, intention.*

From the result of mediation effect analysis, individual benefits, social benefits, and willingness-to-pay have the indirect effect on purchase intention through attitude. Furthermore, self-competence, convenience, and environmental literacy influence purchase intention significantly through perceived behavioral control. However, the results revealed that the mediatory role of the subjective norms is not effective. Therefore, the government should not regulate or promote through subsidies, but rather encourage consumers to voluntarily share their knowledge and experiences through their social networks and participate in the green wave as partners of the government instead of as passive helpers.

## Conclusion

In this study, we analyzed the success factors for green consumption by using theory of planned behavior. According to Kalafatis et al. (1999), theory of planned behavior is a reliable and foundational model of intention to purchase green products. Therefore, this study adopts theory of planned behavior model to provide an additional perspective into the antecedents of purchase intention toward green products. The empirical findings revealed that consumers’ attitude and perceived behavioral control strongly and sustainably impact purchase intentions for green products through individual and social benefits and self-competence, convenience, and environmental literacy, respectively; however, subjective norms do not affect purchase intentions. Consumers’ attitude represents individual preferences, whereas perceived behavioral control represents the social pressure on the green consumption. Thus, these empirical results reveal that public regulation of the green consumption is unsustainable in Taiwan. To establish the effect missing in subjective norms, environmental responsibility should be replaced with strong partnerships between the private and public sectors.

For transformation toward sustainable growth, the governance of subjective norms should be complemented with the public–private partnership. However, in most Asian countries, government leadership is overemphasized and thus, the green wave presents a type of moral hazard in the private sector. Even if there is a wide consensus on green consumption on the Internet and among the public, economic transformation toward an advanced green economy is challenging if individual consumers understand green policy regulations as individual, intimate demands. In 2015, the Korean government initiated a nationwide emission trading scheme; however, the government fixed its carbon-reduction targets at substantially lower levels than originally proposed because of the complaints from the participating companies, which resulted in lack of governance for sustainable development. Therefore, without the support of the private sector, government- or society-driven sustainable transformation in developing countries is challenging. The harmonized management of the public–private partnership should therefore be implemented carefully and in detail to achieve performance-oriented governance.

The research limitations and future research directions are presented as follows. First, due to a single source that is applied to measure the whole measurement items, the possibility of a common method bias should be assessed. Although Harman’s single-factor test was adopted to test for common method bias, a more detailed research design should be applied to evaluate the existence of common method bias. The second limitation is to ignore the assessment of differences among various types of users and actual behaviors toward green products. Future work may attempt to execute a perspicacious analysis including moderation and mediation effect examination if necessary. Finally, the generalizability of the findings might be cautiously made, for this study was conducted in a single-area setting (i.e., Taiwan). Future studies could use this model in various cultural settings, and compare and contrast the findings among them. Additional effort across different areas or cultures should be discussed for generalizing the research findings.

## Data Availability Statement

The raw data supporting the conclusions of this article will be made available by the authors, without undue reservation, to any qualified researcher.

## Author Contributions

AR, J-JY, and S-WC contributed to research design. YM and L-CC performed the sample collection and data analysis. AR, YM, and S-CC wrote the manuscript. YM, S-CC, and L-CC developed the original idea for the study. AR and S-WC conducted the research design. All authors contributed to the article and approved the submitted version.

## Conflict of Interest

The authors declare that the research was conducted in the absence of any commercial or financial relationships that could be construed as a potential conflict of interest.

## Questionnaire

### Individual Benefits

(Bhattacherjee, 2000; Kotler and Keller, 2006):

• Environmental-friendly products can avoid harmful gas (such as: environmental-friendly coating)
• I think the environmental-friendly products with high economic efficiency (such as: hybrid) helps me save spending for a long time.
• A bonus inspired me more willing to do recycling (such as: recycle bonus)

### Social Benefits

(Mainieri et al., 1997):

• Use environmental-friendly products can reduce carbon emissions.
• Use environmental-friendly products is a hand to the society.
• In order to the future of human sustainable you should use environmental-friendly products

### Willingness-to-Pay

(Ozanne and Vlosky, 1997; Wu and Hsing, 2006; Abeliotis et al., 2010):

• Environmental-friendly products with higher sale price is acceptable (such as: pure natural detergent)
• I have higher identity of the environmental label products with higher price (such as: contains no lead)
• Organic food can reduce the burden of the body or higher price is acceptable

### Environmental Responsibility

(Hines, 1984; Simmons, 1991):

• I will buy environmental-friendly products on account of it helps to reduce damage to environment.
• I will buy environmental-friendly products on account of it can promote resource recycling.
• I will buy environmental-friendly products on account of it beneficial to the sustainability of the earth

### Electronic Word-of-Mouth

(Bickart and Schindler, 2001; Hennig-Thurau, 2004; Hanson and Kalyanam, 2007):

• When I saw the collection point, I will try to concentrate of plastic bottle/batteries to there (such as: convenience stores collection points)
• Global environmental pollution problem is increasingly serious.
• I’m more and more concern myself with environmental pollution in Taiwan

### Values

(Rokeach, 1973; Mainieri et al., 1997):

• I usually search the relevant information of environmental-friendly products on the internet.
• If anyone have falsely accused to some environmental-friendly products, I will put forward or reply my positive views.
• I will refer to others’ experience who used environmental-friendly products as mine reference to purchase

### Self-Competence

(Roth, 1992; Fraj and Martinez, 2006):

• I think the environmental label product is worth buying even though the cost is high.
• I think the products which use the environmental-friendly material is worth buying even though the cost is high.
• If most people think that use environmental-friendly products can be beneficial to the living environment, I will be willing to do

### Convenience

(Chen, 2007):

• I’ll be influenced by TV advertising to buy environmental-friendly products.
• I will buy environmental-friendly products on account of my friends are using
• I will buy environmental-friendly products on account of my family are using

### Environmental Literacy

(Roth, 1992; Culen and Volk, 2000; Farmer et al., 2007):

• I have a deeper cognition on the natural environment after I used the environmental-friendly products (such as: natural lotion - soapberry)
• I have enough knowledge to identify with low polluting products
• I’m an environmental literacy citizen

### Attitude

(Ajzen, 1985, 1991, 2002; Kotler, 1997; Blackwell et al., 2001)

• I will use environmental-friendly products on account of its easy to obtain (such as: natural crystal soap)
• Environmental protection information awareness programs to facilitate the public have a better understanding of environmental protection (such as: environmental protection information affixed to the bus, the MRT)
• I will use environmental-friendly products because of the convenience of shopping through the Internet

### Subjective Norms

(Ajzen, 1985, 1991):

• I think it may impact on the environment to use of polluting products
• I think it may reduce the impact on the environment to use of environmental-friendly products
• I think there are close relationship between ecological balance and the survival and sustainable development of mankind

### Perceived Behavioral Control

(Ajzen, 1985, 1991; Han et al., 2010):

• I will buy environmental-friendly products on account of satisfying experience
• I will buy reusable environmental chopsticks on account of bad experience to use disposable chopsticks (such as: pungent taste, bamboo chopsticks are not polished)
• I will buy environmental-friendly products because of the entrenched environmental concepts

### Purchase Intention

(Ajzen, 1985, 1991; Chatzisarantis and Hagger, 2005):

• I’m willing to buy environmental-friendly products in the coming year
• If prices are no significant differences with others, I may purchase environmental-friendly products
• If quality are no significant differences with others, I may purchase environmental-friendly products
